# Drone Noise Emission Characteristics and Noise Effects on Humans—A Systematic Review

**DOI:** 10.3390/ijerph18115940

**Published:** 2021-06-01

**Authors:** Beat Schäffer, Reto Pieren, Kurt Heutschi, Jean Marc Wunderli, Stefan Becker

**Affiliations:** 1 Empa, Swiss Federal Laboratories for Materials Science and Technology, 8600 Dübendorf, Switzerland; reto.pieren@empa.ch (R.P.); kurt.heutschi@empa.ch (K.H.); jean-marc.wunderli@empa.ch (J.M.W.); 2 BeSB GmbH Berlin Schalltechnisches Büro, 12203 Berlin, Germany; s.becker@besb.de

**Keywords:** drones, UAV, UAS, multicopter, noise emission, noise effects, annoyance, perception

## Abstract

The number of operations of Unmanned Aerial Vehicles (UAV), commonly referred to as “drones”, has strongly increased in the past and is likely to further grow in the future. Therefore, drones are becoming a growing new source of environmental noise pollution, and annoyance reactions to drone noise are likely to occur in an increasing share of the population. To date, research on drone noise emission characteristics, and in particular also on health impacts, seems scarce, but systematic overviews on these topics are missing. The objective of this study was to establish a systematic literature review on drone noise emissions and noise effects on humans. The paper presents the methodology of the systematic reviews performed separately for noise emission and noise effects, assembles current literature, gives an overview on the state of knowledge, and identifies research gaps. Current literature suggests that drone noise is substantially more annoying than road traffic or aircraft noise due to special acoustic characteristics such as pure tones and high-frequency broadband noise. A range of open questions remains to be tackled by future studies.

## 1. Introduction

Unmanned Aerial Vehicles (UAV) or unmanned aircraft systems (UAS), commonly referred to as “drones”, are a relatively new transportation noise source in the environment. While initially mainly used and developed for military purposes, they are becoming increasingly important also in civil applications, even more so as they have a very wide spectrum of applications (see, e.g., [1]), ranging from leisure time activities to commercial applications (delivery services) to medical, public safety and scientific purposes. In the USA, for example, there were 1.32 million small UAS (sUAS ≤ 25 kg) registered for leisure time purposes and 0.385 million sUAS for professional applications in 2019 [2]. These numbers were forecasted to increase by 12% and 115% to 1.48 million and 0.83 million in 2024 for leisure time and professional applications, respectively (baseline forecasts in [2]). Thus, while noise exposure of the population is currently small, it is likely to become an important issue in the future.

The fast development of drone operations makes legislation and standardization necessary. The ANSI Standardization Roadmap of 2020 [3] identified that no specific standards for drone noise are available to date, and high priority was given to that topic (see p 246 of the Roadmap). However, to establish drone noise standards and legislations to address and minimize noise issues, among others, a profound knowledge of acoustical characteristics depending on operational procedures as well as related health impacts on the population is necessary. Limiting the noise impact on the population at an early stage is crucial, even more so because the opinion of the population on drones still seems to be forming [4]. Thus, the acceptance might develop rather advantageously or disadvantageously for drones, depending on the perceived relative importance of benefits or (noise) risks by drones [1,4].

To date, research on acoustical characteristics of drones and in particular also on the impact of drone noise on humans still seems quite scarce. While some general overviews on drones are available [5,6], only two overviews focus on noise [7,8]. Further, we are not aware of any systematic reviews according to the PRISMA Statement (Preferred Reporting Items for Systematic Reviews and Meta-Analyses) [9] on this topic.

The objective of this study was to establish two separate systematic literature reviews on drone noise. The reviews aim at giving an overview on (i) measurement practices and noise emission characteristics such as sound power levels and directivity, and (ii) noise effects on humans. The literature reviews were conducted according to the PRISMA Statement [9]. Based on the general procedure, the detailed protocols were specifically adapted for the reviews, as described below. Besides establishing the state of knowledge, we identify knowledge gaps to motivate possible future research.

## 2. Systematic Review of Studies on Noise Emission Characteristics: Methodology

### 2.1. Research Question and Study Selection Criteria

The current systematic review of studies on noise emission characteristics aims at answering the following research question:

*Which experimental methods can be used to measure the acoustic emissions of drones and convert them into an empirical model, and which emission data are already known and documented?*

Studies are included in the analysis that potentially make statements on the measurement methodology for drones or provide indications for the formulation of empirical emission models or list emission data. Only studies that consider the drone as an overall system are of interest. Studies specifically examining only partial sources, like single propellers, are excluded. In the analysis of the identified studies, the proposed measurement methods and modeling approaches are discussed with respect to (i) required infrastructure, (ii) measurement effort, (iii) level of detail of the emission model that can be derived, and (iv) expected model accuracy. Thereafter, the available emission data are compiled.

### 2.2. Formalism of the Database Search

We included the following electronic literature databases in the literature review (parentheses indicate the searched fields): Web of Science (via EndNote) (title/keywords/abstract), Scopus (title/keywords/abstract), ScienceDirect (title/keyword/abstract), Ingenta-Connect (title/keywords/abstract), Google Scholar (title), conferences: Euronoise, International Congress on Acoustics ICA, International Congress on Sound and Vibration ICSV, Inter-Noise. No proceedings were found for the FAI International Drones Conference and Expo. The Quiet Drones 2020 symposium had been postponed to a future date at the time of the database search, but was searched later (cf. Section 2.4). All publications until date of database search were included in the literature search.

For the literature search, the following string was developed and used:

(“drone*” OR “quadcopter” OR “multirotor” OR “multicopter” OR “UAV” OR “UAS” OR “unmanned air*” OR “unmanned aer*”) AND (“acoustic” OR “sound” OR “noise” OR “auralization*”)

The first part of the string specifies the noise source type and the second part the aspect of interest.

The database search was performed between 17 August and 19 August 2020. The search yielded the following hits (“database: number of hits”), including duplicates:

- Web of Science (via EndNote): 1891
- Scopus: 3594
- ScienceDirect: 193
- Ingenta-Connect: 151
- Google Scholar: 481

This resulted in a total of 6310 records, all of which were imported into EndNote.

### 2.3. Title–Abstract and Full-Text Screening of the Database Search

From the original collection, duplicates were automatically removed in EndNote using the <Find Duplicates> function, leaving 4987 entries for the subsequent manual screening. However, a larger number of entries were still listed twice; these were removed during the subsequent title–abstract screening process. The triage also excluded entries that were not relevant because they:

- Refer to a partial aspect of the sound generation (e.g., blade investigation) and not to the drone system as a whole;
- Have nothing to do with the acoustic emission of drones (e.g., when the term “noise” refers to noise in a signal or when the drone is used as a tool to investigate some other aspect);
- Refer to drone noise captured by microphones flying with the drone;
- Deal with the localization or identification of drones by means of microphone arrays without being directly related to the determination of emission data;
- Investigate the noise reduction of drones; or
- Use the term “drone” in a different context.

After this screening process, 72 studies remained as presumptively relevant. The full text of 67 references could be obtained. Three missing studies are conference papers of the American Acoustical Society for which no written versions exist, one reference concerns the study of a drone powered by a combustion engine published in a Russian journal, and one reference is a conference paper that is presumed to be covered by other publications included in the collection.

In the full-text screening of the remaining 67 records, 46 references turned out to be irrelevant, either because only certain aspects but not the drone as an overall system were examined, the measurement set-up was not generalizable or not specified in enough detail, or military flying objects were described. This left 21 relevant articles.

### 2.4. Other Sources

A manual internet search did not yield any additional hits. However, the list of relevant references was subsequently supplemented by a Quiet Drones 2020 conference paper, a VOLPE report on drone measurements (ICAO homepage), and an own journal article.

### 2.5. Summary of the Literature Search

**Figure 1:**
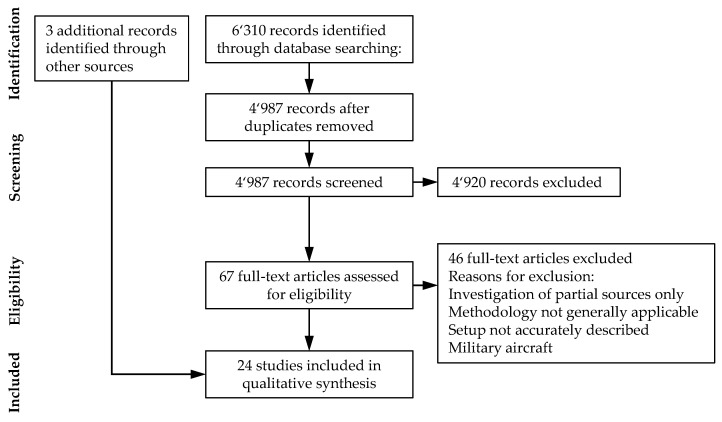
PRISMA flow diagram of the systematic review on drone noise emission characteristics.

Figure 1 summarizes the literature search using the PRISMA flow diagram. The systematic procedure resulted in a total of *n* = 24 documents for the subsequent review.

### 2.6. Data Extraction

The key data of the included studies were extracted into an overview table with information on drone model, maneuver, laboratory/field measurements, microphone setup, and emission data. Usable noise emission values were identified in the studies and converted into comparable acoustical quantities. The table is presented in Section 4.

## 3. Systematic Review of Studies on Drone Noise Effects on Humans: Methodology

### 3.1. General Approach and Methodology

For the literature review on noise effects, we adapted the published study protocol PROSPERO 2013 CRD42013006004 by Seidler, et al. [10] to our review purpose, which had been used, e.g., by Weihofen, et al. [11] and Hegewald, et al. [12]. This protocol was considered a suitable starting point, as it had been used for a systematic review on the related topic “Systematic review: environmental aircraft noise and non-auditory health complaints and diseases” [10].

In a first step, a database search was performed (Section 3.3 and Section 3.4), and thereafter, other sources were additionally searched for relevant literature (Section 3.5).

### 3.2. Research Question and Study Selection Criteria

The current systematic review of studies on noise effects on humans aims at answering the following research question:

*Are individuals exposed to environmental drone noise at an increased risk of noise annoyance and/or acquiring other non-auditory health effects?*

In answering the research question, we paid attention to the following aspects:

*May level corrections be identified to account for stronger noise effects compared to reference noise sources such as road traffic?*

*Which acoustic metrics or psychoacoustic parameters are particularly suitable to predict the noise effects of drones?*

*Do the non-acoustic moderators visual–acoustic interactions and context (or acceptance) affect noise effects of drones?*

Here, context is understood as a factual and situational background that is initially given to the subjects of a psychoacoustic study, e.g., what sound sources are involved or whether the study is on rescue flights or commercial drone operations. Context can strongly moderate the effects of drone noise on study subjects or a population in general. For example, noise of rescue operations is more likely to be accepted than of private flights [1,4], which may in turn result in higher acceptance and reduced noise annoyance. Besides this, individual characteristics such as age, gender, noise sensitivity, or attitude toward the noise source may strongly moderate noise effects (e.g., [13]). These characteristics are considered here as additional moderators, independent of context. Finally, visual-acoustic interactions can moderate noise effects (e.g., [14]). For drones, safety and privacy concerns were shown to play a role [1,4], which might be increased by visible drones [15].

As the focus of the current review is on psychoacoustic studies, the research questions explicitly mention the effect dimension noise annoyance, but also consider health effects in general. Based on the above research question, we specified the study eligibility criteria, i.e., inclusion and exclusion criteria, using the Population–Exposure–Outcome (PEO) characteristics (e.g., [12]) as well as further criteria, as shown in Table 1. The inclusion criteria were further specified using the search string, while the exclusion criteria were refined for screening (see below). Note that the inclusion criteria were defined very generally, as we expected that not many psychoacoustic and/or health effect studies on drone noise are currently available. The aim, therefore, was to provide as complete an overview as possible.

### 3.3. Formalism of the Database Search

We included the following electronic literature databases in the literature review (parentheses indicate the searched fields): Web of Science (title/keywords/abstract), Scopus (incl. Embase) (title/keywords/abstract), MEDLINE (via PubMed) (title/keywords/abstract), PsycInfo (über ProQuest) (all fields), Ingenta-Connect (title/keywords/abstract), Psyndex (via PubPsych) (key words/title/author/source/ISSN/ISBN/abstract), Google Scholar (title), conferences: Inter-Noise, Forum Acusticum (incl. e-Forum Acusticum 2020), Euronoise, International Congress on Acoustics ICA, International Congress on Sound and Vibration ICSV, Noise-Con, ICBEN Congress, DAGA, Quiet Drones Symposium. Prior to the conferences search we checked whether they were covered by the other databases. Only conferences not covered were separately searched. All publications until date of database search were included in the literature search.

For the literature search, the following string was used, which had been developed for Web of Science and was adapted to the other databases accordingly:

(“drone*” OR “quadcopter” OR “multirotor” OR “multicopter” OR “UAV” OR “UAS” OR “RPA” OR “unmanned air*” OR “unmanned aer*”) AND (“acoust*” OR “sound” OR “noise” OR “psychoac*” OR “loud*” OR “sharp*” OR” rough*” OR “tonal*” OR “tone*”) AND (“annoy*” OR “disturb*” OR “affective” OR “psychol*” OR “stress” OR “risk” OR “health”)

The first part of the string specifies the noise source, the second part the acoustic and psychoacoustic characteristics (loudness, sharpness, roughness, fluctuation strength, and tonality; see [16,17]), and the third part the health outcomes.

The database search was performed between 24 August and 8 September 2020, with the exception of the Quiet Drones Symposium (search date: 24 November 2020) and the e-Forum Acusticum 2020 (28 December 2020), as these conferences were held later. The database search yielded the following hits (“database: number of hits”), including duplicates:

- Web of Science: 350;
- Scopus (incl. Embase): 669;
- MEDLINE (via PubMed): 48;
- PsycInfo (via ProQuest): 113;
- Ingenta-Connect: 24;
- Psyndex (via PubPsych): 0;
- Google Scholar: 3;
- Conferences: 1.

This resulted in a total of 1208 records, all of which were imported into EndNote.

### 3.4. Title–Abstract and Full-Text Screening of the Database Search

Title–abstract screening was performed in Rayyan QCRI (https://www.rayyan.ai/) (accessed on 29 May 2021), a web application for systematic reviews [18]. Rayyan QCRI helps identifying duplicate records. Further, it supports efficient screening with keywords as potential inclusion and exclusion criteria (Table 1), which are marked green and red, respectively, in title and abstract. Further, reasons for the exclusion of publications can be indicated, which are summarized in an overview statistics.

As potential inclusion criteria we chose the following: drone, drones, unmanned, UAV, UAS, sound, noise, annoy, annoyance, health, population, human. As potential exclusion criteria we chose the following: disturbance(s), stress, review, RPA, animal, animals, reviews, whales, rats, editorial, fish, transgenic, in vitro, porcine, canine, mouse, rat. Note that while the key words “stress” and “disturb*” were contained in the search string (see above), they were mostly used in contexts that are not related to noise effects. References were excluded due to the following reasons: no health effects, no drone topic, full proceedings (not individual articles), wrong publication type, and review publication.

In total, 370 duplicate records were removed. After the title–abstract screening, 10 articles were retained as potentially relevant, the full texts of which were then obtained.

In the full text screening of the remaining records, six further articles were excluded. Reasons for exclusion were that the articles did not investigate noise effects and/or that they only gave an overview of already published research studies without presenting new results data. Four relevant articles remained.

### 3.5. Other Sources

In addition to the electronic database search, an internet search was performed with the Google search engine in the time period from 24 to 26 November 2020, using the search string specified in Section 3.3 as well as various simplified strings such as “drone annoyance”. We searched for psychoacoustic studies in general, as well as specifically for NASA, Swedish and French studies as we expected research activities there. In addition, the reference lists of publications included in the full-text screening (see above) were searched for further relevant articles. Finally, the German journal *Lärmbekämpfung*, which is not covered by the electronic databases, was screened for relevant articles. Potential records were directly subjected to title–abstract and full-text screening to assess their suitability. A total of four additional articles were thus added to the database.

### 3.6. Summary of the Literature Search

**Figure 2:**
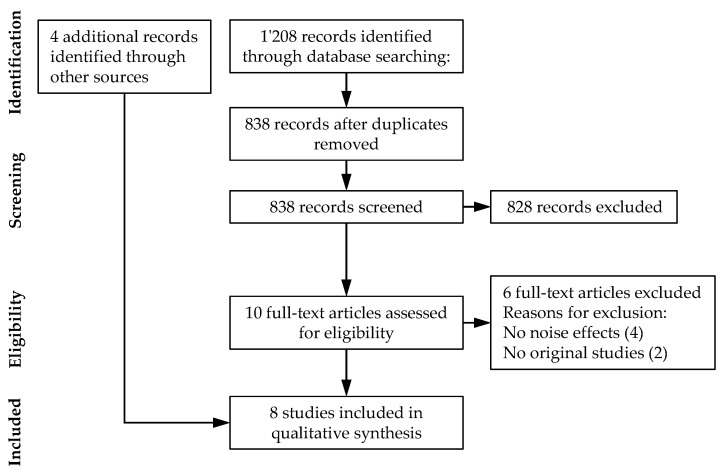
PRISMA flow diagram of the systematic review on noise effects of drones.

Figure 2 summarizes the literature search using the PRISMA flow diagram. Due to the small number of remaining documents included in the analysis (*n* = 8) which addressed different aspects of drone noise effects, a quantitative meta-analysis was not possible. Therefore, a qualitative synthesis was conducted on the literature.

### 3.7. Data Extraction and Quality Rating of Studies

The key data of the included studies were extracted into an overview table. The overview table was developed starting from the tables with summaries of study characteristics given in [12] and adapted to the purpose of the present study. It includes, where available, information on the drone model, category (e.g., quadcopter), weight, flight control during sound recordings (manual vs. autopilot), flight conditions, other environmental noise sources considered, study region, design, quality score, study population, and generation and rendering of stimuli (for laboratory experiments). Further, the table specifies information on whether information/context was given, health outcomes and their assessment methods, whether acceptance, audio-visual aspects, and/or background noise were studied, (psycho)acoustic metrics, experimental sound pressure level range, psychoacoustic sound pressure level difference, data analysis methods, main results, keywords, and assessment of the strengths and limitations of the studies. Based on the latter, the study quality was scored as good (++), medium (+), or poor (-). We refrained from a detailed quality assessment based on criteria according to SIGN (Scottish Intercollegiate Guidelines Network 2004) or CASP (Critical Appraisal Skills Programme 2004/2006) (see, e.g., [12]), as a quantitative synthesis was not feasible. Likewise, risk of bias (e.g., Cochrane risk-of-bias) was not assessed in detail, but where information on the latter was available (e.g., regarding context), it was considered in the study assessment. An abbreviated version of the overview table is given in Section 5.

## 4. Drone Noise Emission Characteristics: Results

A total of 24 studies were included in the systematic review on noise emissions of drones (Figure 1). Table 2 lists the studies and reports the considered types of drones, the flight maneuvers, the microphone arrangements, and the reported noise emission data. Note that the analyses refer exclusively to studies of pure multicopters. Fixed-wing aircraft or mixed forms are not addressed. Below, the measurement methods used are first discussed (Section 4.1) and usable emission values are identified and converted into comparable characteristic values (Section 4.2). Thereafter, the vertical source directivity (Section 4.3) and special sound characteristics of drones are contemplated (Section 4.4).

### 4.1. Measurement Methods for Noise Emissions of Drones

Of the 24 relevant studies, 14 describe laboratory investigations, and 10 studies document field measurements (Table 2). The laboratory measurements are divided in standard sound pressure laboratory measurements and special laboratory measurements. The former are setups that directly measure the sound pressure generated by a fixed or hovering drone, typically at multiple positions. The latter are measurements that determine the drone’s emission indirectly, e.g., by using a microphone array or an acoustic camera, or that require a special laboratory environment.

#### 4.1.1. Standard Sound Pressure Laboratory Measurements

The Regulation 2019/945 by EU [24] describes a test procedure for drones and specifies the maximum allowable A-weighted sound power levels, *L_W_*_,A_, for various drone classes. The procedure prescribes the operating condition “hovering at a height of 50 cm above reflective ground” and determines the radiated sound power by assessing the sound pressure on a hemispherical measurement surface. Except from the floor, walls and ceilings must be absorbent or sufficiently far away from the measurement setup to avoid contributions from reflections. The advantage of this procedure is that it follows an established methodology, but only limited directional information for an emission model can be derived from the data obtained.

Herreman [26] determines the radiated power of a drone in an anechoic chamber using 20 sound pressure microphones set up on a spherical surface with radius 0.9 m around the fixed drone. By choosing different electrical power settings, different operating conditions of the drone can be investigated. With knowledge of the power required for a particular flight maneuver, the laboratory value can be assigned to that maneuver. The measurement effort is large with 20 sensors, but a detailed emission model results. Only smaller drones can be measured with the setup, and the transferability of the data to outdoor operation still remains to be proven.

Heutschi, et al. [27] determine the emission of multicopters in a semi-anechoic chamber using five sound pressure microphones arranged at different elevation angles at a distance of 1.5 m from the source center. The drones are operated at different rotational speeds by attaching various weights in the hovering state, resulting in a rotational speed dependent emission model. The measurement effort is relatively small with five sensors, but the directivity in the lower hemisphere is modeled with only four data points. The assumption that the acoustic emission of a particular drone depends exclusively on the rotor speed still has to be confirmed.

In an anechoic chamber as well, Intaratep, et al. [29] investigate the radiation of a fixed drone with a single sound pressure microphone. The microphone position captures a radiation angle of −40° with 1.3 m lateral to and 0.77 m below the rotor plane. With this minimal setup, too little information on acoustic emission is obtained to formulate an emission model.

Using 10 sound pressure microphones arranged on a vertical quarter circle with radius 1.5 m in an anechoic chamber, Klug, et al. [31] measure the vertical radiation pattern in 10° resolution. The drones are fixed and operated at different electrical power settings. The measurement effort remains moderate with 10 sensors and allows for a sufficiently fine resolution of the directivity. The proposed fixing of the drone has the disadvantage that the controllers of the control electronics operate at an unrealistic state.

Mobley [32] uses 20 sound pressure microphones distributed on a spherical surface of radius 1.8 m in an anechoic chamber to measure the acoustic radiation from fixed drones with different electrical power settings. With 20 sensors, the instrumental effort is relatively large. A proof of transferability to real flight operations in real atmosphere is pending.

For the determination of the sound power of drones, Papa, et al. [33] measure the sound pressure on a hemisphere with radius 1.2 m in an anechoic chamber. Here, the drone is fixed and operated at different electrical power settings. The measurement effort is relatively low with 5 or 10 sensors. However, it is not clear why the microphone positions are chosen in the upper hemisphere, although the drone is usually expected to fly over the receiver points of interest.

Tinney and Sirohi [38] determine the sound radiation of a drone in an anechoic chamber using eight microphones placed either at different distances or elevation angles. The drone is operated fixed at different speeds.

#### 4.1.2. Special Laboratory Measurements

Cheng and Herrin [23] use an intensity probe to sample the sound pressure and sound velocity on a closed surface around the hovering drone to estimate the sound pressure at an arbitrary position in the far field using the Kirchhoff–Helmholtz integral. This procedure appears to be very laborious and does not generate any obvious added value compared to standard sound pressure measurements.

Using an array consisting of 72 microphones laid out on the floor of a large anechoic chamber (10.5 m × 8.4 m × 7 m), Fattah, et al. [25] investigate the sound radiation from hovering and slow-flying drones. Although the dimensions of the laboratory make even forward flight operation possible, the velocity must remain so small, in the order of 1 m/s, that hardly any measurement closer to reality than in the hovering condition is achieved. The microphone array allows sound pressure level maps to be made in the array plane. Although the instrumental effort is very large, the directivity can only be recorded in a limited solid angle, so that a complete emission model is not obtained. Zhou, et al. [42] present a similar setup. The measurements are conducted in the same anechoic chamber that allows slow forward flight. Again, a 72-element horizontal microphone array is used, but here supplemented by eight vertically arrayed microphones to study the radiation dependence on the elevation angle.

Using an acoustic camera, Kloet, et al. [30] investigate partial sound sources on a drone. The measurement allows a qualitative assessment of the different components, with the rotors in first place, followed by the motors. The data cannot be directly converted into sound power or sound pressure level at a given distance.

Based on the assumption that sound radiation arises exclusively by the interaction of the drone with the surrounding air, Putzu, et al. [34] place the drone in a laboratory in which an airflow is generated by a large number of small fans. Thus, with the drone fixed with respect to laboratory coordinates, forward flight can be “simulated.” In contrast to a conventional wind tunnel, the so-called WindShaper also allows the generation of inhomogeneous wind fields. An important limitation in the measurement of sound radiation is the background noise of the airflow generation.

Following a similar concept, Zawodny and Pettingill [40] study the fixed drone in a low-noise wind tunnel and thus establish conditions for forward flight despite the drone being stationary. This setup allows specific investigations of various aspects of sound generation.

#### 4.1.3. Field Measurements

Alexander and Whelchel [19] and Alexander, et al. [20] describe sound pressure measurements from drones hovering and slowly flying at 3.2 m/s over grassy ground. Five microphones are placed directly on a 1 m acoustically hard ground plate. The microphones are arranged on a line perpendicular to the forward flight path to cover different radiation angles. The sound exposure level of the flyby is used as a signal feature to characterize the noise emission. The measurement effort is moderate, but no explicit emission directivity can be derived from the presented evaluations of the integral sound energy for whole flybys.

Besnea [21] describes field measurements of hovering and forward-flying drones using a microphone array. Although this sensor technology allows source localization, the correspondingly high effort for source strength characterization does not seem to offer an advantage over single sound pressure microphones.

Cabell, et al. [22] describe sound pressure measurements of various hovering and forward-flying drones, where four microphones are placed on a 43 cm plate each. The microphones are located on a line perpendicular to the flight path, and the A-weighted maximum sound pressure level is measured. By inverting the propagation loss (back-calculation to the source) and comparing the maximum levels at the different microphones, an angle-dependent emission model can be found. However, capturing lateral radiation angles requires large microphone distances, which makes back-calculation uncertain and cannot cover all possibly relevant radiation angles.

A large, field-deployable 180-element microphone array for measuring drone flights is presented by Humphreys, et al. [28]. In addition to source localization, the microphone array also allows synchronously analyzing the sound pressure signals generated at different radiation angles. The instrumental effort is very high.

Kloet, et al. [30] measure a drone hovering at different heights with a sound pressure microphone at 1 m above grassy ground. The microphone signal is characterized using the A-weighted sum level. The problem of interference between direct and ground-reflected sound is not addressed. The measurement setup in this form is not suitable for emission model generation.

Senzig and Marsan [36] demonstrate emission characterization using a drone flyover at 150 m. The resulting sound pressure is measured with a microphone on a ground plate, converted to 400 feet (122 m) distance from the source, and reported as an A-weighted maximum sound pressure level. The resulting emission model has no angular dependence.

In a similar study, Senzig, et al. [37] and Read, et al. [35] compare the sound pressure signals of drone flyovers at a microphone installed at 1.2 m above ground and a microphone placed directly on a ground plate. Here, the flyover events are characterized by the maximum and sound exposure levels. As expected, the levels measured on the plate are consistently higher than at 1.2 m height. The A-level differences found are typically 2 dB. The resulting emission model has no angular dependence.

For different drone operating conditions, Treichel and Körper [39] show A-weighted sound pressure levels resulting from multiple microphones at different heights above reflective ground. With simultaneously recording eight microphones, horizontal and vertical directivity data are collected for the hovering condition. With moderate measurement effort, data are collected that can be translated into a directional emission model. However, since the microphones were not in every case far from the ground, the data are spectrally distorted as a result of interference between direct and reflected sound.

Using an array of 40 boundary microphones, Zhang, et al. [41] study the hovering and forward flight of a drone at different altitudes. The focus of the study is on beamforming, which enables acoustic localization of the drone.

### 4.2. Noise Emission Levels of Drones

In a separate analysis, the studies listed in Table 2 were reviewed with regard to sufficiently well documented and comprehensible measurement data. Since the investigations are very heterogeneous and differ strongly with respect to the measurement set-up, drone models and operating states as well as the reported noise metrics, a rigorous selection had to be made. Only studies were included here that (i) describe the investigated drone model sufficiently well at least by specifying its mass, (ii) investigate “hovering” and/or “forward flight” as the operating state of the drone, and (iii) describe the geometry of the drone and microphone position sufficiently accurately.

A total of 10 studies met these criteria. Table 3 and Table 4 list the collected data for hovering and forward flight, respectively. From the drone’s perspective, the reported radiation angle describes elevation with respect to the horizon formed by the rotor plane. A negative sign indicates a downward radiation; −90° is the direction vertically downward.

In order to make the measurement data of the different studies comparable, the A-weighted sound pressure level (*L_p_*_,A,1m,−30°_) at a distance of 1 m under free-field conditions and at a radiation angle of −30° with respect to the rotor plane was introduced as a parameter to characterize the acoustic emission. To transform the different measurement data into this emission value, the following normalizations or conversions were made:

- Translation dB(Z) into dB(A): For a typical drone emission spectrum, the two sum levels differ only slightly, so where necessary they were set equal.
- Geometrical spreading: Point source far-field behavior in the form −20log(*d*).
- For a pressure zone microphone mounting on a ground plate, a sound pressure doubling or level increase by 6 dB is assumed with respect to free field.
- The drone is assumed to emit 3 dB more in the A-weighted level vertically downwards than at −30° [27].
- The emission level is estimated from the sound power level by: *L_p_*_,A,1m,−30°_ = *L_W_*_,A_ − 11 dB.
- The amplification effect of the ground for a microphone at a height of 1.0 to 1.2 m is assumed to be 1 dB(A) above grassland and 3 dB(A) above hard ground.

**Figure 3:**
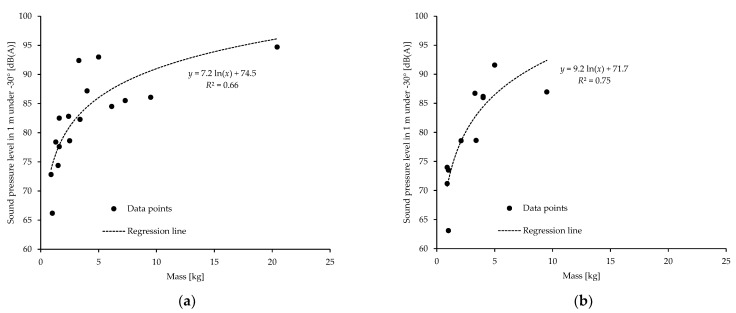
Noise emission of multicopters (data points and regression models) in forward flight (**a**) and hover (**b**) as a function of take-off mass: Free-field emission values of multicopters as A-weighted sound pressure level at a distance of 1 m for a radiation angle of −30° as a function of the drone mass.

The emission levels thus derived were analyzed with respect to vehicle mass and maneuver. Figure 3 shows the results for the two operating conditions hover and forward flight. The noise emission is slightly higher for forward flight than for hover. For drone masses of up to about 5 kg, i.e., the range where data is available for both conditions, an average level difference of some 0–3 dB(A) results.

### 4.3. Vertical Source Directivity

**Figure 4:**
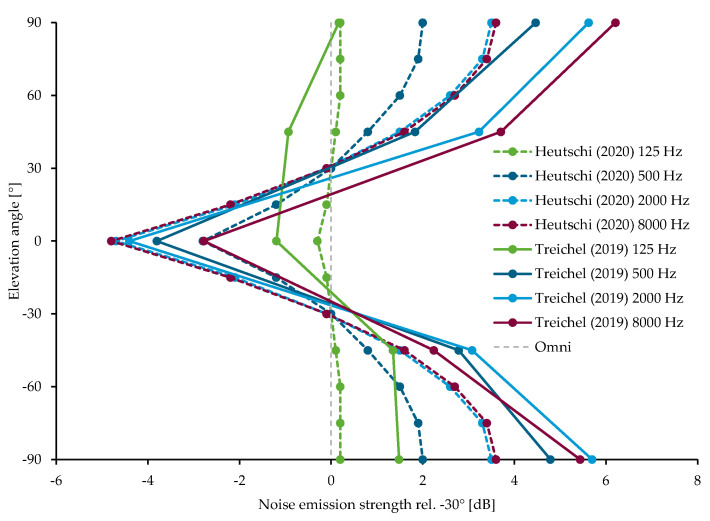
Vertical noise source directivity patterns of multicopters at different frequency bands (data: Heutschi, et al. [27]; Treichel and Körper [39]). The vertical line (“Omni”) indicates an omnidirectional radiation, i.e., monopole.

Two studies provide spectral and angle-dependent information on the source directivity of multicopters. Treichel and Körper [39] present the mean of three different drone models measured outdoors, while Heutschi, et al. [27] present a generic spectral directivity derived from laboratory measurements on five different drone models operated at different rotational speeds. For the overview shown in Figure 4, all data have been referred to a radiation in direction −30° (below horizon) with respect to the propeller plane. The data shows that in the mid to high frequency range the directivity effect between horizontal and vertical radiation direction amounts to about 10 dB, indicating that vertical directivity must be considered in an emission model.

### 4.4. Excursus: Spectral Noise Emission Characteristics of Drones

In this section, we provide some exemplary data on spectral noise emission characteristics of drones obtained from the author’s own measurements at Empa [43], as this aspect was not part of the systematic review.

**Figure 5:**
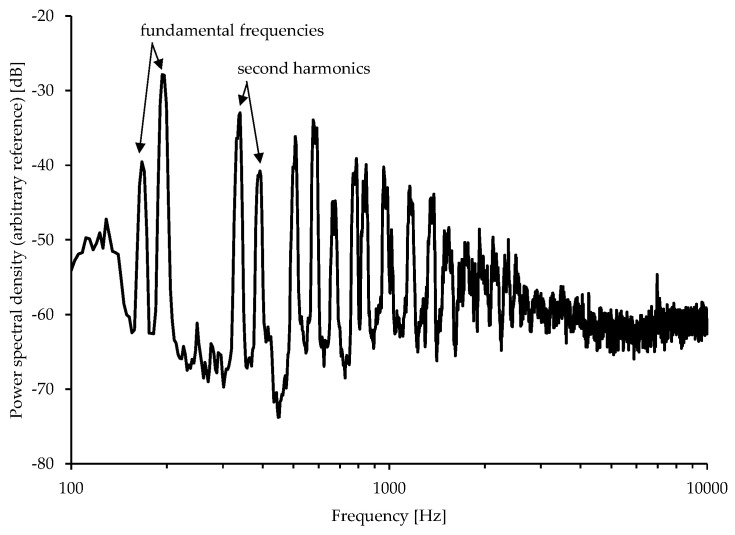
Narrowband spectrum of a hovering multicopter DJI Mavic 2 Pro measured in the laboratory [43].

The spectral characteristics of the acoustic emission from multicopters include low- and mid-frequency tonal and harmonic components coupled to the rotor speed, and high-frequency broadband noise whose power density decreases weakly with frequency (see, e.g., [8,44]). Figure 5 exemplarily shows the power spectral density of a hovering DJI Mavic 2 Pro multicopter measured in the laboratory under −30° obtained from the authors’ own measurements at Empa. The tonal components related to one harmonic appear in pairs with slightly different frequencies. This indicates the operation of the four propellers in two groups.

**Figure 6:**
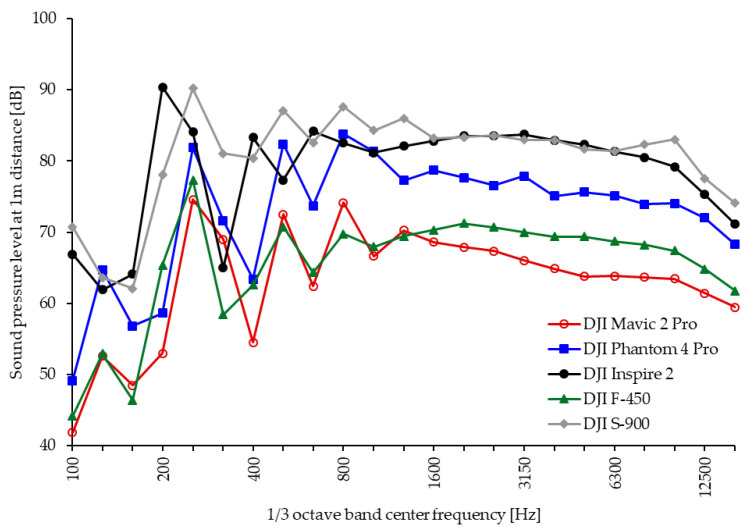
Noise emission strengths in 1/3 octave bands of different multicopters operating at maximum power [43].

In a spectrally coarser resolution of 1/3-octave bands, the spectral peaks and dips progressively disappear with increasing center frequency and the correspondingly increasing analysis bandwidth. Figure 6 shows the 1/3-octave band spectra of the sound pressure level at a distance of 1 m at −30° for various drone models operated in the laboratory at maximum power obtained from the authors’ own measurements at Empa [43].

## 5. Drone Noise Effects on Humans: Results

A total of eight studies were included in the systematic review (Figure 2). Table 5 summarizes the study characteristics with the major key data of the studies (abbreviated version of the full table as described in Section 3.7).

To date, only a few laboratory experiments on the acute, short-term effects of acoustic or visual–acoustic drone stimuli on humans, and no field studies on long-term effects of drones are available. With the exception of the study by Rizzi, et al. [45] on a fixed-wing propeller aircraft with distributed electric propulsion high-lift system, all studies assess the noise effects of multicopters (mainly quadcopters; in two studies also octocopters). So far, the maneuvers hovering [44,46,47,48,49] and straight flyover [44,45,48,50] were studied under different payloads, but not starts and landings. Except for Rizzi, et al. [45], who used auralizations, and Callanan, et al. [46], who used real drones hovering during the experiments, the studies’ acoustic stimuli were based on sound recordings. One of the studies, presented in a webinar, shows only the results of an initial pilot experiments and the concept of how the actual laboratory study should be conducted [51], while another study shows results of a calculation exercise with different “Psychoacoustic Annoyance” models [44]. Nevertheless, given the small number of available studies, the latter two studies are also included here. So far, mainly the acute, short-term, perceptual, (psychoacoustic) noise annoyance under laboratory conditions has been studied. This health outcome determined in the laboratory, referred to as “noise annoyance” in the following, needs to be distinguished from the psychoacoustic annoyance based on model calculations [16] as well as from the long-term noise annoyance in the field (e.g., [52]). Except for [48], noise annoyance was the subject of all studies. Besides this, individual studies also addressed other aspects of noise effects, as detailed below.

### 5.1. Drone Noise Annoyance

Little surprising, drone noise annoyance strongly depends on the sound pressure level [45,46,49,50]. In this respect, drones are no exception from other transportation noise sources. 

**Figure 7:**
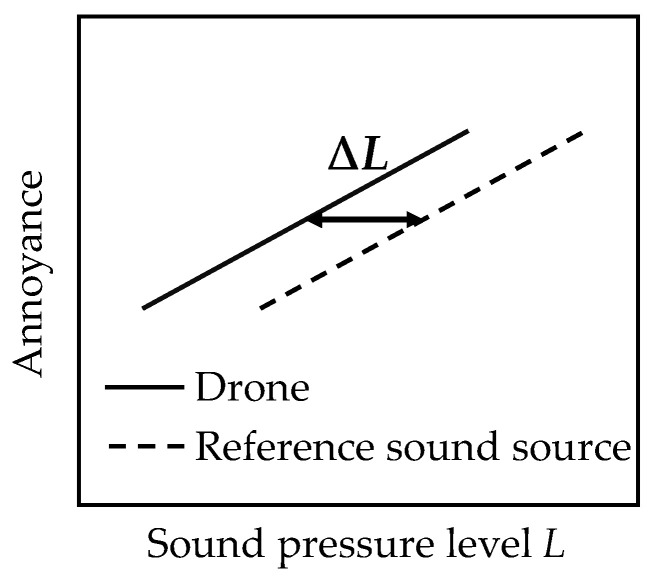
Illustration of the psychoacoustic sound pressure level difference (∆*L*): hypothetical exposure–response curve for annoyance as a function of sound pressure level of a drone and a reference sound source, and ∆*L* as the horizontal shift of the two curves.

Besides this, five studies confirm an increased annoyance potential of drones as compared to other transportation sources. Christian and Cabell [50], in their much-cited study (the first one on psychoacoustic effects of drone noise), compared the annoyance effects of multicopter flyovers with road vehicle pass-by events. On average, drones were substantially more annoying than road vehicles at a given sound pressure level. This source-specific annoyance difference may be quantified by means of a psychoacoustic sound pressure level difference (∆*L*) (Figure 7). It corresponds to the horizontal shift of exposure–response curves (here, for annoyance) and quantifies at what sound pressure level a reference noise source is equally annoying as (in this case) drones. In [50], drones were found to be equally annoying as road vehicles with a 5.6 dB higher single event level (*L*_AE_), i.e., ∆*L* = 5.6 dB. Other values resulted for other noise metrics (*L*_CE_, EPNL, L5) (Table 5), with *L*_AE_ being most strongly related to annoyance. Gwak, et al. [49] found that the annoyance to hovering multicopters was significantly higher than to a starting jet aircraft, with ∆*L* = 4–10 dB in sound pressure level ranges of around 70–80 dB(A). Besides this, ∆*L* depended on drone size. Torija, et al. [47] studied the effect of a hovering quadcopter in different urban soundscapes. They found that the presence of the drone, compared to the same soundscapes without the drone, significantly increased annoyance even in situations with increased road traffic noise (*L*_Aeq_ ≈ 65 dB). The same annoyance increase would be evoked by an increase in the background noise level alone, i.e., without the drone, of 6 dB or more, and in quieter soundscapes, this effect would even be much more pronounced. Further, Torija, et al. [44] in their calculation exercise found that the Psychoacoustic Annoyance of a quadcopter computed with the models of Fastl and Zwicker [16], Di, et al. [53] and More [54] was higher than that of road vehicles and in particular also jet aircraft. Finally, the study by Torija and Li [48] on the preference (an “inverse indicator” of annoyance) of different sound sources found that the preference of a quadcopter was significantly lower than that of jet aircraft and road vehicles.

The significant differences in annoyance and the large values of ∆*L* at a given A-weighted sound pressure level indicate that common noise metrics such as the *L*_AE_ or *L*_Aeq_ cannot fully represent the effect of drones compared to other noise sources: the stronger a noise metric is related to a health impact, the smaller ∆*L* will become [50]. This is where psychoacoustic parameters come into play. Psychoacoustic parameters reflect the human perception of various acoustic characteristics [16]. A good overview of this can be found, for example, in [17]. The parameters include loudness, sharpness, roughness, fluctuation strength, and tonality [16,17]. Gwak, et al. [49] found that drone noise annoyance was primarily influenced by sharpness, loudness, and fluctuation strength. Further, particularly sharpness, but also loudness, led to annoyance differences between drones and jet aircraft. Indeed, in a second laboratory experiment, the authors decreased the unpleasantness of drones by reducing sharpness and fluctuation strength of the stimuli with signal processing. However, the psychoacoustic parameters failed to explain the differences between the larger drones and the smallest one. According to the authors, one reason might have been the lower tonality of the smallest drone, which, however, was not studied further [49]. In fact, Torija and Li [48] found that the preference of different sounds largely depended on tonality as well as the interaction of loudness with sharpness. Rizzi, et al. [45] developed a psychoacoustic model based on loudness, roughness, and tonality to predict annoyance to the electric fixed-wing aircraft over a wide range of design and operational parameters and correspondingly different acoustic signatures. Finally, also the calculation exercise by Torija, et al. [44] shows that psychoacoustic parameters can be used to predict the (expected) increased annoyance potential of drones.

### 5.2. Effect of Design and Operation of Drones on Noise Annoyance

Currently, only few studies systematically study the influence of design and operation of drones on annoyance. Gwak, et al. [49] found that annoyance increased with increasing drone weight or size (∆*L* ≈ 6 dB between a small and a large drone weighting 0.11 kg and up to 11 kg, respectively). However, the (unquantified) lower tonality of the smallest drone might have affected the results [49] (see above). Rizzi, et al. [45] found for the electrical fixed-wing aircraft that annoyance increased with the number of propellers (6–12–18). This was expected as the (auralized) sound power increased with the number of propellers, because their rotational speed was held constant in the auralizations [45]. Time-varying effects also somewhat influenced annoyance (details see [45]), but not revolutions per minute (RPM) differences between propellers. The remaining studies (Table 5) did not examine the effects of design and operation. They either used only one specific drone type (studies by Torija, et al.) or did not account for differences between types [46].

During operation, the relatively slow flight speed of drones compared to other means of transport seems to particularly contribute to the increased noise annoyance. Christian and Cabell [50] found differences in the *L*_AE_ of up to 8 dB for quadcopter flyovers with a speed of 5 m/s at altitudes of 20, 30, 50, and 100 m (and corresponding longer duration), but no differences in the annoyance ratings. Accordingly, subjects commonly stated that they rated sounds that seemed to “loiter” as more annoying than other sounds. Furthermore, Torija, et al. [47] found that the annoyance ratings of different soundscapes hardly changed over a sound pressure level range of about 6 dB in the presence of the drone. As suggested in [50], this effect might be accounted for with a “loitering penalty” based on geometric considerations, which might explain most of the psychoacoustic level difference ∆*L*. However, it might still be reasonable to separately account for specific acoustic characteristics such as tonality.

In addition to operating drones at a higher speed, it could be reasonable to concentrate drone operations in corridors along noisy environments (e.g., major roads) to benefit from a certain masking effect. The louder the noise environment, the lower the emergence of the drone noise from background noise and consequently the (additional) noise annoyance to drone noise might be [47,51]. Similar findings were reported by other studies, e.g., on the (partial) masking of wind turbine noise by increased background noise [55]. However, the number of studies on this topic is still quite scarce, and the effects of combined multiple noise sources on health outcomes should be studied in more detail (e.g., [56]).

### 5.3. Further Health Outcomes

Besides annoyance, some studies investigated further health outcomes. Begault [51] presented the concept of “Annoyance—Blend—Detection”, with the idea that drone noise should blend into soundscapes and not dominate them, while operating non-detectable drones is hardly realistic. (An analogous concept, “Annoyance—Noticeability—Audibility”, was proposed by NASA [7].) Blend is a compromise between annoyance and detection at a signal-to-noise ratio where drones do not dominate other ambient sound sources. This concept might allow defining an “acceptability” threshold, above which the noise will be perceived as intrusive.

Torija, et al. [47] investigated the effect of a hovering drone in various audio-visual situations of urban parks at different distances from roads on annoyance, subjectively perceived loudness and pleasantness. The authors studied the effect of acoustic stimuli alone as well as visual–acoustic stimuli, using video recordings of the parks with and without the drone. The presence of drone noise negatively affected all outcomes. This was mainly due to the resulting *L*_Aeq_ increase, but also due to acoustic characteristics of the drone. The visual information affected the different outcomes to varying degrees. While it hardly influenced the subjectively perceived loudness, it was linked to slightly lower annoyance and substantially higher pleasantness (+47%) of the stimuli. Overall, subjective loudness was mainly determined by the *L*_Aeq_, annoyance equally by the *L*_Aeq_ and the presence/absence of the drone, and pleasantness depended most strongly on the *L*_Aeq_ and equally (more weakly) on the presence/absence of the drone and the visual information.

Callanan, et al. [46] studied the impact of drone noise on communication, subjectively perceived loudness and annoyance in a warehouse situation as a potential work environment with drones. Speech signals were played with a loudspeaker, without and with a real drone hovering close to the loudspeaker. Two quadcopter types were subsequently used to represent a “loud” (~83 dB(A) close to the drone) and “quiet” (~68 dB(A)) drone noise situation. As expected, annoyance and loudness were found to increase with increasing drone noise level, while subjectively perceived and objectively assessed communication (speech comprehension tests) were the more impaired, the lower the signal-to-noise ratio of the speech signal to drone noise was. For communication, also the reverberation of the room may have played a role, but this aspect was not studied [46].

## 6. Discussion

To our best knowledge, the current systematic literature review provides the first overview on drone noise emission (emission measurements and characterizations) and the effects of drone noise on humans. The current literature gives a consistent image of drones as a special new environmental noise source and their effects on humans.

### 6.1. Drone Noise Emission Characteristics

#### 6.1.1. Current Measurement Methods

To date, a wide range of experimental setups is available, from laboratory to field experiments. While laboratory experiments offer high control, but at the expense of transferability to the field, field experiments exhibit a high degree of realism but lower control and thus external disturbance factors. The used methods range from single microphone measurements to determine level-time histories, sound exposure or maximum levels by several microphones to special experimental setups such as microphone arrays to reveal individual noise sources or directivity, and wind tunnel measurements to simulate “real” outdoor conditions under high laboratory control.

The measurement methods used to record the sound emitted by drones—both in the laboratory and in the field—can be divided into the two classes: (i) single- and independent-multichannel microphone arrays and (ii) array microphone arrangements with phase reference between sensors.

Most studies assume a non-omnidirectional radiation characteristic that is pronounced at least in the vertical plane. This directional characteristic is either captured with several microphones covering different elevation angles, or by an entire overflight event evaluated with one microphone and characterized by the sound exposure or maximum level. The dependence of the acoustic emission on the electrical power or the rotational speed is investigated in several studies in the laboratory and converted into an emission model. On this basis, the emission under real flight conditions can be estimated.

In the majority of measurements, ground reflections are either largely suppressed (virtually all laboratory measurements) or then made easily correctable by boundary layer mounting and the resulting sound pressure doubling.

Microphone arrays can be used for source localization by means of beamforming, or—more relevant in our context—to generate time-dependent sound pressure level maps in the ground plane. By that, two-dimensional directivity effects become apparent.

Compared to conventional laboratory measurements, investigations in the wind tunnel offer significant advantages and enable a widely realistic operation of the drone. However, the equipment required is very expensive and the usable frequency range is limited due to the high background noise of the wind tunnel facility.

#### 6.1.2. Noise Emission Characteristics

The most important aspects of drones as acoustic sources can be summarized as follows. The emission strength primarily depends on the drone model and payload, as well as on the operating state or the flight maneuver. Various studies prove that drones exhibit a pronounced vertical angular emission directivity, while in the horizontal plane, a uniform radiation is expected for symmetry reasons. 

Theoretical considerations on the balance of forces and experimental observations suggest that there is a correlation between operating condition and rotor speeds. It might thus seem feasible to formulate a rotational speed-dependent emission model and then, using knowledge of the required maneuver-dependent speed, to estimate the emission for any given operating condition. However, this approach is hindered by the fact that in horizontal flight, forward motion is only made possible by different rotational speeds of the front and rear rotors. Further, since the interaction between the rotors also plays a role in sound generation, this case cannot be easily estimated from a speed emission model typically obtained for hovering. Nevertheless, as Figure 3 shows, noise emission during hover and forward flight is not too different, with level differences of 0–3 dB, so that hover measurements (e.g., in the laboratory) may still be used as a first proxy for emission characteristics of forward flight until better data is available.

The current review yielded no emission data for the flight operations climb and descent. While climbing represents a flight condition that is broadly comparable to hovering, except for elevated RPM levels, descent maneuvers feature different sound characteristics. As a consequence of unsteady airflow conditions, often a strong variation of RPM levels of the different rotors occurs, leading to fluctuating sound pressure levels with constantly varying pure tone components. As descent maneuvers are in addition typically flown at low vertical speeds, they involve considerable event durations. Consequently, this flight phase might cause considerable annoyance reactions for residents nearby landing areas and should therefore be studied in more detail and evaluated separately. 

#### 6.1.3. Emission Model and Data Acquisition: A Proposition

Taking the aforementioned acoustic properties into account, a drone emission model must meet the following demands: (i) show the spectral emission strength for different operating conditions, (ii) describe and reproduce the vertical radiation characteristics, and (iii) express the emission as radiation into the free field so that a simple coupling to a sound propagation model for the calculation of the exposure at any receiver point is possible.

Translating these emission model requirements into a measurement procedure possesses various challenges. First, the radiation pattern can be determined most reliably for a fixed-position or slow-moving drone. When a microphone is flown over fast, a large angular range is captured if the geometry is suitable, but the typical angular changes per unit time are so large that adequate signal averaging times are not obtained. Thus, the elevation angle-related estimates become uncertain. Second, the back calculation of a microphone signal to the source must, in the case of free field propagation, include geometric divergence, air attenuation and ground effect. With appropriate knowledge of geometry, air temperature, and humidity, the first two propagation effects can be considered with good accuracy. The inversion of the ground effect, however, requires the ground properties and an appropriate ground reflection model. Finally, in order to facilitate the comparability of future studies, a minimum set of acoustical parameters and their exact determination should be defined. Here, maximum or sound exposure levels are suitable for flyovers, and equivalent continuous sound pressure levels (Leq) for hovering.

With the above background, a pragmatic emission model and corresponding measurement concept could make the following assumptions. The emission model distinguishes the four operating states hovering, climbing, descending, and level flight with “typical” forward speed. An identical relative vertical directivity is assumed for all operating states. Thus, in a first step, the directional characteristic for the hovering operating condition could be measured in the laboratory or outdoors with microphones at high altitude or in boundary arrangement on the ground. Thereby, the drone can either be in fixed position or in freely hovering conditions. Operating and varying RPM in fixed position is easier to achieve. However, great attention has to be given to an accurate leveling of the drone. If not done, the automatic stabilization control of the drone is likely to produce inconsistent RPM values for the individual rotors. In that aspect, free hovering is more realistic. There, varying operational conditions, i.e., RPM values, can be achieved by adding different payloads. Care has again to be taken to attach them in a way that the center of gravity of the drone is not altered. The microphone effort can be minimized if the drone hovers at different heights. In a second step, the operating states of climb, descent and level flight would be measured, with the signal at a single drone direction sufficing as an anchor point with the directional characteristics assumed to be known. On that basis, an angular independent, potentially frequency dependent, correction compared to hovering conditions can be derived. The microphone would again advantageously be installed either high above ground or in boundary mounting.

In any case, an exact temporal synchronization between the acoustic recording and the system registering the drone position is required, with position accuracy depending on the geometry. Distances that are too small should be avoided to ensure that the drone can be regarded as a point source and that the air movement caused by the drone rotors does not generate any non-negligible wind noise at the microphones. The analysis of the microphone signals would have to provide at least 1/3-octave band sound pressure levels for the emission model. For the determination of tonality and other parameters, the audio signals should also be recorded. In conclusion, to establish such source models, measurements with several (5–10) microphones are necessary. A single microphone is not sufficient for that purpose, while microphone arrays and/or wind tunnel are not compulsory.

### 6.2. Drone Noise Effects on Humans

#### 6.2.1. Noise Effects and Implications

To date (database search early September 2020; other sources late November 2020), only few psychoacoustic laboratory studies and no field surveys on drone noise effects are available (Table 5). This finding is consistent with other current overviews [5,7,8]. Accordingly, ITF [6] recommends to support research on perception of and annoyance to drone noise. Current research primarily focused on noise annoyance (Table 5). Despite the small number of studies, the results provide a fairly consistent picture on annoyance effects of drone noise on humans.

Available literature suggests that drone noise is substantially more annoying than road traffic or aircraft noise at the same level. This shows that available exposure–response curves, e.g., to predict the probability of high annoyance to aircraft noise [57], are hardly transferable to drone noise. The increased annoyance to drone noise could be accounted for with level corrections (or, alternatively, stricter limit values for drone noise in environmental guidelines/legislation). However, currently available studies do not allow reliably defining such correction terms. First, the data basis is too small (two laboratory studies only [49,50]). Second, similar psychoacoustic level differences were found for both road traffic and aircraft noise in the order of 5 dB [49,50], although aircraft noise is substantially more annoying than road traffic noise [57,58]. Quite similar level differences might have resulted due to the “anchoring effect” of laboratory studies. Each experiment has a different reference frame, since the reference to the real (noise) environment is missing. The differences obtained in the two studies are therefore not directly comparable. Third, level differences determined in the laboratory should be confirmed in field studies.

The increased annoyance to drones is linked to special acoustic characteristics, in particular pure tones and high-frequency broadband noise. These characteristics are not sufficiently represented by common noise metrics such as the *L*_Aeq_ or the Effective Perceived Noise Level (EPNL) to accurately predict noise effects. Therefore, the aircraft noise certification scheme, based on EPNL, is hardly appropriate for drones [59]. Here, psychoacoustic parameters may be better suited. However, also they may hardly account for all potentially annoying characteristics of drones, since the annoyance is also affected by non-acoustic factors such as the slow flight speeds (“loitering”). Nevertheless, psychoacoustic parameters are promising to assess drone noise as well as to support acoustical optimization of drones using a “perception-influenced design” [60]. In fact, acoustically optimized drones (reduced overall emissions, high-frequency components and tonality) in the trial operation of a drone delivery service in Australia resulted in only a few noise complaints despite thousands of deliveries [61]. In general, noise issues of drones should be treated with high priority to facilitate sufficient public acceptance. Eißfeldt, et al. [4] found that noise concerns were a decisive factor for attitude towards drones. While noise concerns were still quite low compared to concerns about crime and privacy, people who had actually heard drone noise before were more concerned [4], so that noise might become a growing issue in the future.

Two important aspects of drone noise effects remain unexplored. First, context is likely to be an important moderator for annoyance. As an example, the use of drones for public safety, rescue and scientific purposes is much more accepted than for leisure time or commercial purposes [1,4], which might in turn affect annoyance. However, no study provides information on whether/what context was given to the subjects, although Christian and Cabell [50] apparently did not provide any information (the authors note that very few subjects recognized drone noise as such), while the subjects in the study of Torija, et al. [47] who were presented videos of a hovering drone will have recognized the noise source. Thus, to what degree context might affect annoyance remains unanswered. Second, visual information might moderate noise annoyance. Indeed, presenting videos was found to reduce annoyance [47], potentially by distracting from the annoying noise. Similar results were also found, e.g., for wind turbine noise [14]. In contrast, no studies on the effect of drone visibility on annoyance are available (in the videos of [47], the drone was always visible). Visible noise sources were previously found be linked to increased annoyance (e.g., [14,62]). For drones the same is expected, even more so due to privacy and safety concerns [1,4,15]. Further, the effect of source visibility may depend on the environment (e.g., [63]). To what extent this applies to drones is currently unknown.

#### 6.2.2. Limitations of the Studies of the Systematic Review

All studies considered in the systematic review (Table 5) are characterized by a scientifically sound study design. Nevertheless, certain study limitations should be considered when interpreting the systematic review.

Generally, the ecological validity of the available literature on drone noise effects is limited, as only laboratory studies with a limited number of subjects (25–50) are available. Inferring from short-term noise annoyance in the laboratory to long-term noise annoyance in the field is therefore afflicted with increased uncertainty (e.g., [52]).

In addition, some studies have specific limitations. First, one study did not randomize playback order of the stimuli [46]. Hence, bias due to serial position effects [64] might have affected the results. Second, several studies do not account for subjects’ repeated (and thus interdependent) observations in the statistical analysis [45,46,50]. Third, [46] did not use an ICBEN scale for annoyance, while [50] used the ICBEN 5-point scale but “translated” the results into a 10-point scale, both of which reduces the comparability with other studies. Fourth, none of the studies disclosed what study information was given to the subjects. Thus, the potential influence of context remains unaccounted for. Fifth, one study found that annoyance increased with drone size [49]. Here, however, tonality might have played a role, which was not studied, so that the effect of drone size remains uncertain. Finally, not all studies used ecologically highly valid acoustic stimuli. The most realistic stimuli were used by the NASA studies [45,50], where drone flybys or overflights were auralized using a three-dimensional reproduction system. Further, one study reproduced stimuli obtained by dummy head recordings via headphones [49], while another study used real hovering drones for their experiments [46], which are both highly realistic. Two other studies, in contrast, used mono recordings for the hovering operating condition [47,48], which limits ecological validity due to a lack of realistic spatial representation and thus immersion. Furthermore, the recordings were made in the laboratory with a fixed drone, which might not fully represent the “real” hovering condition due to potentially different noise characteristics of fixed compared to non-fixed drones. In addition, several studies changed the sound pressure level of parts of the stimuli by level adjustment instead of propagation filtering, which reduces ecological validity [44,47,48,49,50]. The above limitations were also considered in the assessment of study quality (Table 5).

#### 6.2.3. Knowledge Gaps and Future Research

While the focus of current literature is on annoyance, a number of open questions remain. Annoyance should thus be further pursued, even more so as it is the most frequent adverse health effect besides sleep disturbance [65]. To that aim, laboratory studies and, as soon as real (trial) operations are run, field surveys should be conducted to complement the laboratory studies with ecologically more valid results, to reliably estimate the noise effects of drones in the population.

The following aspects are of particular interest (see also the recommendations of [7,8]). First, comparisons of drones to other transportation noise sources are still scarce. Comprehensive laboratory studies including drone, helicopter (as the vehicle most similar to drones), jet, and possibly propeller aircraft flyovers as well as road vehicle pass-by events in a single experiment are desirable to establish exposure–response curves and source-specific level corrections. Second, systematic studies on the influence of design and operation of drones using different multicopters, weight classes and flight conditions at varying payloads and speeds, with a special focus on slow flybys (“loitering”) and descent, are missing. Third, systematic variation of (psycho)acoustic characteristics of drones could help developing a psychoacoustic model for drone noise annoyance. Such systematic studies could reveal most effective annoyance reduction potentials by design, operation and acoustical optimization (“perception-influenced design” [60]). Fourth, the impact of drone noise on (urban) soundscapes (cf. [47]) should be further explored. Drones may severely (negatively) affect soundscapes in future. This necessitates profound understanding of the impact of drone noise on soundscape perception (beyond noise annoyance) for different soundscape contexts, in order to establish timely mitigation measures [66]. Here, soundscape research (e.g., [67,68,69]) is a promising approach to complement acoustic and psychoacoustic studies. Finally, additional aspects such the influence of context, visual–acoustic interactions and soundscape on drone noise annoyance, but also on blend and detection, as well as the effects of drone noise on health outcomes other than noise annoyance should be further explored.

### 6.3. Bringing Both Reviews Together: A Step towards Strategic Noise Mapping?

The current literature review on drone noise emission is a step towards bringing drones into the framework of strategic noise mapping according to the Environmental Noise Directive (END) [70]. A pragmatic drone noise emission model is outlined in Section 6.1.3. Such a model can be coupled with a suitable sound propagation model, preferably CNOSSOS-EU compliant as prescribed for road, railway, and industrial sources (END Annex II [71]). Given that sufficient information of (future) drone operators is available, such as drone types, number and time of movements, air routes, and flight trajectories, this would allow establishing noise maps using common noise indicators such as the *L*_den_ or *L*_night_, as well as to quantify the number of affected persons above certain noise exposure levels.

To assess the situation regarding health impacts on the population, however, the suitability of common noise indicators for drones is uncertain (cf. Section 6.2.1). For example, specific drone noise characteristics might not be sufficiently covered by a single noise indicator but might require drone-type specific penalties (e.g., for loitering and/or tonality). In addition, source-specific noise limits need to be established. This, however, requires a range of further laboratory and in particular also field studies as identified by the current literature review on noise effects, to allow for quantitative meta-analyses of drone noise effects on humans, similar to the meta-analyses established for the WHO 2018 Environmental Noise Guidelines [72].

## 7. Conclusions

In this study, two separate systematic reviews based on the PRISMA statement were performed, namely, on measurement practices and noise emission characteristics of drones, and on noise effects on humans. The review on emission characteristics revealed that the source strength primarily depends on the drone model and payload, as well as on the operating state or the flight maneuver. Drones exhibit a pronounced vertical emission directivity but radiate rather uniformly in the horizontal plane. An emission model should account for the correlation between operating condition and rotor speeds. The literature of drone noise effects on humans is still very scarce. Nevertheless, the current literature provides a fairly consistent picture, suggesting that drone noise is substantially more annoying than road traffic or aircraft noise due to special acoustic characteristics, in particularly pure tones and high-frequency broadband noise. However, various aspects, such as the factual and situational context, soundscape, and audio-visual interactions, all potentially affecting noise effects, remain largely unexplored. There is a need for future research to characterize drones as noise sources and explore their effects on humans to inform standards and legislation, with the aim of minimizing the negative impacts of future drone operations on the population.

## Figures and Tables

**Table 1:** Inclusion and exclusion criteria according to Population–Exposure–Outcome (PEO) characteristics and further criteria.

Category	Inclusion Criterion	Exclusion Criterion
Population (P)	Population (humans, i.e., children and adults)	Animals
Exposure (E)	Drone noise/sound	Other environmental noise sources
Outcomes (O)	Noise annoyance, general health	-
Other	-	Review articles, newspaper articles, letters, etc.; references to full conference proceedings instead of individual conference articles.

**Table 2:** Studies included in the systematic review on drone noise emission characteristics.

Study	Drones	Maneuver	Lab/Field	Microphones	Emission Data
Alexander and Whelchel [19]	DJI Matrice 600 Pro, Hexa, 15.5 kg	Hovering, slow flyover (3.2 m/s)	F	5 mics on 1 m ground plates on grass on line perpendicular to flight path	*L*_E_ [dB(Z)] and power spectral density
Alexander, et al. [20]	DJI Matrice 600 Pro, Hexa, 15.5 kg	Hovering, slow flyover (3.2 m/s)	F	5 mics on 1 m ground plates on grass on line perpendicular to flight path	*L*_E_ [dB(Z)] and power spectral density
Besnea [21]	Various	Hovering, Climb, Forward flight	F	Microphone array	
Cabell, et al. [22]	Various up to 7 kg	Hovering, Forward flight	F	4 mics on 43 cm ground plates on line perpendicular to flight path	*L*_Amax_, Effective Perceived Noise Level (EPNL), spectrograms
Cheng and Herrin [23]	DJI Mavic Pro	Hovering	L	Intensity probe	1/3 octave band sound pressure at 5.5 m
EU [24]	Not specified	Hovering	L	Hemispherical measuring surface according to ISO 3744	Sound power
Fattah, et al. [25]	Quadcopter, 1.4 kg	Hovering, slow flight	L	Microphone array	
Herreman [26]	KittyHawk HDX15,17, GPX SkyKing	Fixed, 10, 50, 60, 70, 80% power	L	20 mics on sphere of radius 0.9 m	Sound power
Heutschi, et al. [27]	DJI Mavic 2 Pro, DJI Inspire 2, DJI S-900, DJI F-450	Hovering, varying payload	L	5 mics on vertical arc with elevations −80 to +30° at 1.5 m	1/3 octave band sound pressure at 1.5 m
Humphreys, et al. [28]	Not specified		F	Microphone array	
Intaratep, et al. [29]	DJI Phantom II	Fixed	L	1 mic at elevation −40° at 1.5 m	Sound pressure dB(A) and power spectral density
Kloet, et al. [30]	Quadcopter	Hovering in field; Fixed in lab with 50% power	L + F	Field: 1 mic 1 m above grass; Lab: Microphone array	Sound pressure dB(A)
Klug, et al. [31]	Little Spyder, Align M480L, FPV-Racingcopter	Fixed, various rpms	L	10 mics on vertical arc in 10° steps at 1.5 m	Sound power
Mobley [32]	KittyHawk HDX15,17	Fixed, various power settings	L	20 mics on sphere of radius 1.8 m	Sound pressure
Papa, et al. [33]	Syma X5C, RC Eye One Xtreme	Fixed, 25 to 100% power	L	11 mics on hemisphere	Sound power
Putzu, et al. [34]	Parrot Bebop 2, DJI Mavic Pro	Airflow simulated forward flight	L	1 mic	Sound pressure
Read, et al. [35]	Yuneec Typhoon, DJI M200, Gryphon Dynamics GD28X	Flyover, hovering, take-off, landing	F	1 mic 1.2 m above ground and 1 mic on ground plate	*L*_Amax_ at 400 feet and *L*_AE_
Senzig and Marsan [36]	DJI Phantom 2, Prioria Hex	Flyover at 150 m	F	1 mic on ground plate	*L*_Amax_ at 400 feet
Senzig, et al. [37]	DJI Phantom 3 Advanced	Flyover at 25, 50, 100 and 200 feet	F	1 mic 1.2 m above ground and 1 mic on ground plate	*L*_Amax_ at 400 feet and *L*_AE_
Tinney and Sirohi [38]	Universal platform, quad, hexa	Fixed, various rpm	L	8 mics sequentially at different elevations and distances	Sound pressure
Treichel and Körper [39]	Not specified models	Hovering, climb, descent, flyover, maneuvering	F	1 mic, or 8 mics for directivity	Sound pressure, sound power
Zawodny and Pettingill [40]	SUI Endurance	Fixed, in wind tunnel to simulate hovering and forward flight at 15.5 m/s	L	Microphone array	Sound pressure
Zhang, et al. [41]	DJI Inspire-1 T600	Hovering and forward flight	F	Microphone array	
Zhou, et al. [42]	DJI Phantom 4	Hovering, climb, descent, forward flight	L	Microphone array on ground and vertical line	

**Table 3:** Acoustical measurement data from literature for multicopters in hover.

Study	Drone Model	Take-Off Mass [kg]	Measurement Values
Alexander and Whelchel [19]	DJI Matrice 600 Pro, Hexa	9.5	Sound exposure level during 14 s on ground plate: −30°, in 18.29 m: 79.2 dB(Z) −45°, in 12.93 m: 83.7 dB(Z) −60°, in 10.56 m: 87.3 dB(Z) −75°, in 9.46 m: 88.7 dB(Z) −90°, in 9.14 m: 89.6 dB(Z)
EU [24]	Not specified	0.9 *	Maximum allowed sound power level as from entry of regulation into force *L_W_*_,A_: 85 dB(A)
EU [24]	Not specified	4.0 *	Maximum allowed sound power level as from entry of regulation into force *L_W_*_,A_: 97 dB(A)
Herreman [26]	KittyHawk HDX17	~5.0	Sound power level *L_W_*_,A_: 102.6 dB(A)
Herreman [26]	KittyHawk HDX15	~4.0	Sound power level *L_W_*_,A_: 97.2 dB(A)
Herreman [26]	SkyKing	~1.0	Sound power level *L_W_*_,A_: 74.1 dB(A)
Heutschi, et al. [27]	DJI Mavic 2 Pro	0.9	Sound pressure level −30° in 1 m: 71.2 dB(A)
Heutschi, et al. [27]	DJI Inspire 2	3.4	Sound pressure level −30° in 1 m: 78.6 dB(A)
Heutschi, et al. [27]	DJI S-900	3.3	Sound pressure level −30° in 1 m: 86.7 dB(A)
Intaratep, et al. [29]	DJI Phantom II	1.0	Sound pressure level −40° in 1.5 m: 70 dB(A)
Kloet, et al. [30]	Self-build	2.1	Sound pressure level −30° in 19 m, 1 m above grass: 54 dB(A)

* Two exemplary masses. Maximum allowed *L_W_*_,A_ are given for masses (*m*) 250 g ≤ *m* < 900 g and 900 g ≤ *m* < 4000 g.

**Table 4:** Acoustical measurement data from literature for multicopters in forward flight.

Study	Drone Model	Take-Off Mass [kg]	Measurement Values
Alexander and Whelchel [19]	DJI Matrice 600 Pro, Hexa	9.5	Sound exposure level during 14 s measured on ground plate for fly-by with 3.2 m/s at 7.5 m height: lateral distance 15.8 m: 79.9 dB(A) lateral distance 9.1 m: 79.6 dB(A) lateral distance 5.3 m: 82.7 dB(A) lateral distance 2.5 m: 84.0 dB(A) lateral distance 0.0 m: 85.3 dB(A)
Cabell, et al. [22]	DJI Phantom 2	1.6	Maximum sound pressure level for fast flyover at 15 m height: 62 dB(A)
Cabell, et al. [22]	Prioria Hex	7.3	Maximum sound pressure level for fast flyover at 15 m height: 65 dB(A)
Herreman [26]	KittyHawk HDX17	~5.0	Sound power level for slow flight *L_W_*_,A_: 104.0 dB(A)
Herreman [26]	KittyHawk HDX15	~4.0	Sound power level for slow flight *L_W_*_,A_: 97.6 dB(A)
Herreman [26]	KittyHawk HDX15	~4.0	Sound power level for fast flight *L_W_*_,A_: 98.2 dB(A)
Senzig and Marsan [36]	DJI Phantom 2	1.6	Maximum sound pressure level on ground plate for flyover at 400 feet: 44.9 dB(A)
Senzig and Marsan [36]	Prioria Hex	2.5	Maximum sound pressure level on ground plate for flyover at 400 feet: 45.9 dB(A)
Senzig, et al. [37]	DJI Phantom 3 Advanced	1.3	Maximum sound pressure level on ground plate for flyover at 25 feet: 69.8 dB(A)
Treichel and Körper [39]	Average over multiple models	~1.5	Maximum sound pressure level at 1.2 m above hard ground for flyover at 5 m: 68.8 dB(A)
Heutschi, et al. [27]	DJI Mavic 2 Pro	0.9	Sound pressure level estimated from hover with payload −30° at 1 m: 72.8 dB(A)
Heutschi, et al. [27]	DJI Inspire 2	3.4	Sound pressure level estimated from hover with payload −30° at 1 m: 82.3dB(A)
Heutschi, et al. [27]	DJI S-900	3.3	Sound pressure level estimated from hover with payload −30° at 1 m: 92.4dB(A)
Read, et al. [35]	Yuneec Typhoon	2.4	Maximum sound pressure level on ground plate for flyover at 400 feet: 50.1 dB(A)
Read, et al. [35]	DJI M200	6.1	Maximum sound pressure level on ground plate for flyover at 400 feet: 51.8 dB(A)
Read, et al. [35]	Gryphon Dynamics GD28X	20.4	Maximum sound pressure level on ground plate for flyover at 400 feet: 62.0 dB(A)

**Table 5:** Studies included in the systematic review on drone noise effects.

Study	Drones; Maneuver	Further Sound Sources	Region; Study Design; Quality	Population	Outcome and Measurement *	(Psycho-)Acoustic Characteristics †	Psychoacoustic Sound Pressure Level Difference
Begault [51]	NASA EVTOL concept; flyover	Different urban soundscapes	USA; study design: n.s., only exp. concept (++)	n.s.	Annoyance, blend, detection: 2-AFC tests; consideration of background sound	Sound level difference (signal-to-noise)	-
Callanan, et al. [46]	2 quadcopters; hovering	Loudspeaker (speech test material)	USA; lab experiment; (+)	*n* = 30 (M = 15, F = 15), 18–34 y; exclusion *n* = 2	Annoyance, loudness, hearing/understanding, ability to listen to voice: 10-point scale; performance: HINT und Alpha-Test	Level-time histories; spectra; *L*_Aeq_	-
Christian and Cabell [50]	3 quadcopters, 1 octocopter; straight flyover	Road vehicles (car, utility van, box truck, step van)	USA; lab experiment; (++)	*n* = 38 (~2/3 M, ~1/3 F), ~18–50 y	Annoyance: ICBEN 5-point scale	*L*_AE_, *L*_CE_; EPNL; L5	∆ *L*_AE_ = 5.6 dB; ∆ *L*_CE_ = 12.8 dB; ∆EPNL = 7.6 dB; ∆L5 = 7.5 dB (drone vs. vehicle)
Gwak, et al. [49]	2 quadcopters, 1 octocopter; hovering	Jet aircraft	South Korea; 2 lab experiments; (++)	Exp. 1: *n* = 50 (M = 35, F = 15), 19–30 y; Exp. 2: *n* = 25 (M = 13, F = 12), 20–30 y	%HA from annoyance: ICBEN 11-point scale; adjectives related to senses and feelings for the sounds: 51-point scale	Spectrograms; spectra; *L*_Aeq_ & further acoustic metrics; L, S, R, FS	∆*L*_Aeq_ ~10 dB (large drone vs. aircraft); ∆*L*_Aeq_ ~6 dB (large vs. small drone); ∆*L*_Aeq_ ~4 dB (small drone vs. aircraft)
Rizzi, et al. [45]	Fixed-wing (electric propulsion); straight flyover	-	USA; lab experiment; (++)	*n* = 32	Annoyance: ICBEN 11-point scale	*L*_A5_; N5, S5, R5, FS5, T5	-
Torija, et al. [44]	1 quadcopter; straight flyover, hover	Road vehicles (car, motorcycle), jet aircraft (A320, A320neo)	Great Britain; calculations (no experiment); (++)	-	“Psychoacoustic Annoyance” models: (1) PA, Fastl and Zwicker [16] (2) PAmod, Di, et al. [53] (3) PAmod for aircraft noise, More [54]	Spectra; N5, S5, R5, FS5, T5	-
Torija, et al. [47]	1 quadcopter; hover	7 urban soundscapes (parks at different distances from roads)	Great Britain; lab experiment (3 parts); (+)	*n* = 30 (M = 16, F = 14), 21–59 y	Loudness, annoyance, pleasantness: (ICBEN) 11-point scale; Consideration of audio-visual interactions and background sounds	*L*_Aeq_; spectra	∆*L*_Aeq_ = 6 dB (annoyance with drone noise vs. background noise only)
Torija and Li [48]	1 quadcopter; straight flyover	Road vehicles (car, motorcycle, moped), jet aircraft (A320, A320neo), reference jet aircraft (B767, B787), [helicopter]	Great Britain; lab experiment: part 2 of [47] (+)	*n* = 30 (M = 16, F = 14), 21–59 y	Ranking in terms of preference: 101-point scale	*L*_Aeq_, *L*_A5_; N5, S5, R5, FS5, T5; EPNL	-

* ICBEN scale: Use of a modified question on short-term annoyance under laboratory conditions, which differs from long-term annoyance in the field; † (Psycho)acoustic characteristics: *L*_A_ (A-weighted level), N (loudness), S (sharpness), R (roughness), FS (fluctuation strength), T (tonality), with 5 (e.g., L5) indicating the 5% percentile.

## Data Availability

Not applicable.
